# Practical approaches to the tasks of preserving autonomy and respecting vulnerability among critically ill adult patients: a narrative review

**DOI:** 10.62675/2965-2774.20250234

**Published:** 2025-03-19

**Authors:** João Gabriel Rosa Ramos, Camila Vasconcelos, Luciana Dadalto

**Affiliations:** 1 Clínica Florence Salvador BA Brazil Clínica Florence - Salvador (BA), Brazil.; 2 Universidade Federal da Bahia Faculdade de Medicina Salvador BA Brazil Faculdade de Medicina, Universidade Federal da Bahia - Salvador (BA), Brazil.; 3 Centro Universitário Newton Paiva Escola de Direito Belo Horizonte MG Brazil Escola de Direito, Centro Universitário Newton Paiva - Belo Horizonte (MG), Brazil.

**Keywords:** Personal autonomy, Vulnerabilities, Ethics, Critical illness, Critical care, Patient care, Cognition, Intensive care units

## Abstract

Respect for autonomy and human vulnerability are bioethical principles that are frequently involved in decision-making dilemmas in the context of critical care. Multiple challenges are involved in the tasks of assessing and respecting the autonomy of critically ill patients with respect to the critical illness in question, patients' cognitive status and the context of intensive care units; furthermore, time constraints and emotional stress complicate decision-making for all stakeholders in this context. In addition, critically ill patients are inherently vulnerable to multiple sources of potential unintended harm. Therefore, clinicians working in intensive care units must develop the skills necessary to acknowledge, assess and mitigate those risks. In this manuscript, we review the literature on this topic. We also propose a practical approach that can help overcome some of those challenges; specifically, we advocate for the adoption of a relational approach to autonomy and shared decision-making, which could help overcome those challenges, thereby promoting more effective and ethical patient care.

## INTRODUCTION

Respect for autonomy is a central value in contemporary medical ethics,^(1)^ and the concept of human vulnerability in health care has been incorporated into this context more recently.^(2)^ These bioethical principles are interrelated^(3,4)^ and may give rise to various dilemmas for health care professionals, such as requests for nonbeneficial treatments and refusal of recommended treatments.^(5,6)^ These dilemmas may be especially pronounced in critical care situations.^(7)^ In this manuscript, we review the literature on the challenges of assessing and respecting autonomy and vulnerability among critically ill adult patients. We also propose a practical approach that can help overcome some of these challenges.

### Autonomy in the context of critical care

Autonomy may be defined as the liberty to act in a manner that is free from coercion,^(8)^ or, as described by Beauchamp and Childress, free from "both controlling interference by others and limitations that prevent meaningful choice, such as inadequate understanding".^(9)^ The exercise of autonomy may be procedurally defined as the ability to self-determine or choose in accordance with one's own best interests.^(8,10)^ Respect for autonomy has been a central principle in bioethics; for example, Article 5 of the Universal Declaration on Bioethics and Human Rights states that "the autonomy of persons to make decisions, while taking responsibility for those decisions and respecting the autonomy of others, is to be respected. For persons who are not capable of exercising autonomy, special measures are to be taken to protect their rights and interests".^(2)^

Limitations of the ability to exert autonomy are common among acutely ill patients. While most patients want to participate in medical decision-making processes,^(11)^ more than 40% of acutely ill patients without mechanical ventilation lack full autonomy, and this proportion is likely higher among patients receiving mechanical ventilation.^(12)^ Moreover, in approximately 75% of cases, this lack of competence is not detected by health care providers,^(12)^ thus indicating that the autonomy of many patients may not be properly assessed and respected.

The limitations to autonomy that have been discussed in the literature are usually internal to the patient; that is, they are to the result of intrinsic characteristics of the individual.^(13)^ Those limitations include the use of psychoactive or sedative drugs, encephalopathy resulting from acute illness, or previous conditions such as cognitive decline among adults or developmental disabilities among children or adult patients.

However, limitations are also frequently external to the patient; that is, they are caused by other individuals or extrinsic constraints that do not result from intrinsic characteristics of the individual,^(13)^ such as the lack of information or communication, social pressure, power asymmetry in patients' relationships with health care providers, disempowerment in terms of decision-making as a result of a lack of resources (including both financial or nonfinancial resources) and acts of coercion or deception on the part of other stakeholders. Those external constraints may even be unintentional, such as when health care providers do not assess patients' understanding accurately or frame their recommendations in ways that may make it exceedingly difficult for patients or their relatives to dissent. As such, both internal and external limitations must be addressed in the context of evaluating patients' competence to exercise autonomy.^(5,13)^

The lack of full autonomy may not be identified because health care professionals often do not obtain genuine informed consent from patients but rather merely obtain "informed assent" or "informed nondissent"^(14-16)^ (Table 1). Consent requires autonomous authorization, which refers to an informed choice regarding a decision that is expressly made by the patient or relatives. Assent is included in Brazilian norms in terms of the expression of the right for information exerted by a person with limited autonomy or competence, such as a child or an individual with cognitive disabilities.^(17,18)^ Therefore, assent is a tool that can help guarantee that individuals whose autonomy is limited are not alienated from the decision-making process. Informed assent, on the other hand, may be viewed as a different concept according to the anglophone literature. It has been described as a form of agreement with a recommendation or the expression of a disposition to accept a treatment by an autonomous individual after that individual has received relevant information.^(16)^ In contexts involving informed assent, after adequate information and communication have been provided, the individual does not expressly make the decision in question but rather agrees to delegate decision-making authority to the clinician. As discussed by Meyers,^(7)^ in contexts involving informed assent, the patient gives permission, whereas in cases involving consent, the patient chooses. Informed nondissent has been viewed as subtly different from informed assent; namely, this case does not involve the delegation of active decision-making authority to the clinician but rather a simple refusal to object to such decisions.^(14,19,20)^

**Table 1:** Definitions and considerations of informed consent, assent, informed assent, and informed nondissent

	Definition	Considerations	Example of a hypothetical interaction with a clinician
Informed consent^(5,7,17)^	An informed choice regarding a decision that is expressly made by the patient or the patient's relatives.	Requires autonomous authorization.	"Due to the deterioration of your condition, we would recommend surgery, which is associated with those risks and benefits. Conservative management is an option but is associated with those risks. I would like to confirm with you that you understand the options and can make an informed choice based on your preferences."
Assent^(7,17)^	The expression of a right for information that is made by a person with limited autonomy or competence.	Can be used as a tool to guarantee that individuals whose autonomy is limited are not alienated from the decision-making process.	"Due to a deterioration of your condition, we would recommend surgery, which is associated with those risks and benefits. Do you agree?"
Informed assent^(16,17)^	A form of agreement with a recommendation or the expression of a disposition to accept treatment by an autonomous individual after that individual has received relevant information.	Following the provision of adequate information and communication, the autonomous individual does not expressly make the decision in question but rather agrees to delegate decision-making authority to the clinician.	
Informed nondissent^(14,15,19,20)^	A model in which the clinician makes a decision and informs the patient/family, after providing them with relevant information, that they will proceed with the decision unless an objection is raised.	This model requires all the elements of informed consent or assent to be present with the exceptions of active choice or agreement. In the critical care literature, this model has usually been discussed in the context of do-not-resuscitate orders and the subsequent fear of appearing to "authorize" the death of a loved one.	"Due to a deterioration in his condition, based on everything we have discussed, we believe that further surgeries or other forms of artificial life support will have no benefits. We will continue treating his symptoms and be with you throughout the process. As you have not objected to this plan, we will proceed with it."

Informed assent or nondissent are common in routine, low-risk situations that lie within the expected standard of care, such as routine physical exams and phlebotomy.^(21)^ However, even in critical situations, informed assent or nondissent may be valid ethical alternatives to informed consent in circumstances in which the practice of informed consent is believed to cause harm, such as in situations involving the possibility of severe psychological damage or when the patient refuses to exert the right of choice.^(14,16,17,19)^ However, even in such circumstances, the clinician must attempt to employ other mechanisms with the aim of respecting patients' autonomy, such as consulting patients' surrogates, before making a decision.^(17)^ Moreover, these circumstances are usually temporary, and the continued use of informed assent, nondissent, or surrogate decision-making may reflect the difficulties experienced by clinicians in their efforts to address a challenging situation. This phenomenon can increase the risk of alienating the patient from the decision-making process and lead to paternalistic decisions that may not respect patients' preferences.

For consent to be valid, the patient must have full capacity to make decisions, relevant information must be disclosed, the decision-maker must have full knowledge and a complete understanding, the choice must be voluntary and a formal authorization must be received.^(5,22)^ The use of objective scores, such as the Mini-Mental Status Examination or the McArthur Competence Assessment Tool for Treatment, may facilitate the assessment of individuals' capacity to make decisions in clinical practice.^(23)^ Such assessment further requires the patient to be able to communicate a choice, to understand the relevant information, to appreciate the situation and its consequences and to reason about the available treatment options.^(23)^ Improper assessment may exacerbate the vulnerability of critically ill patients, thus leading to potentially inappropriate paternalistic decisions. Conversely, improper assessment may give rise to the misleading impression that patients can make fully autonomous decisions when they cannot actually do so. This situation entails the risk of imposing a choice on someone who is unable to make such a decision instead of seeking support from surrogates or other mechanisms of prospective autonomy.^(24,25)^

### Vulnerability in the context of critical care

Vulnerability has been defined in terms of susceptibility to be harmed;^(3,26)^ as such, is a component of the human condition.^(2,27,28)^ However, some individuals or populations may be particularly vulnerable^(2,26)^ as a result of their status, such as the presence of disabilities, diseases or limitations imposed by the various stages of human life, or as a consequence of social, political and environmental determinants.^(2)^ The principle of respect for human vulnerability entails the duty to consider the inherent and unique vulnerabilities of all human beings;^(3)^ accordingly, as discussed by Patrão Neves, susceptibility to be harmed leads to the duty to do no harm.^(26)^ Moreover, the exercise of autonomy and the acquisition of consent does not eliminate vulnerabilities.^(26)^ In this context, isolated autonomy may "remain merely an ideal" as a result of human weaknesses and dependence on external and internal conditions,^(27)^ so that the concealment of the causes of vulnerability may lead to "the making of autonomy a discourse of blaming the victims for their own wounds".^(3)^

Critically ill patients are particularly vulnerable, both because of the power and knowledge asymmetry that characterize their relationships with clinicians and because of their critical illness, which may affect their reasoning and increase their dependency on medical professionals.^(29)^ In addition, critical care scenarios have been reported to entail high risks of exacerbating vulnerability through biases such as ageism,^(30)^ ableism, moral profiling and disparities in terms of gender, race and income.^(31-33)^ For example, patients with disabilities may be viewed as incapable of making decisions without proper assessment; thus, their decisions may be delegated to family members or health care providers. Additionally, many health care organizations do not adapt with the aim of overcoming specific barriers related to cognitive or sensorial disabilities, thereby further impairing communication between health care providers and patients.^(31)^ Moreover, clinicians may not have a proper understanding of specific diseases and disabilities or even fail to assess the impact of biological age properly, thus leading to inappropriate judgments regarding the benefits of treatments or quality of life.^(30)^

Furthermore, baseline medical or social conditions that have been linked with reduced access to health care may lead to more severe acute presentations of disease. When decisions to pursue treatment are based exclusively on the probability of success, this approach may lead to possible stigmatization and a decreased likelihood of treatment among populations that are characterized by baseline disparities, thereby exacerbating injustices.^(33,34)^

In scenarios in which access to life-saving procedures is at stake, clinicians have the power of "causing to live and letting die".^(35)^ In such situations of extreme vulnerability, critically ill patients, especially those who are terminally ill, may have their integrity and dignity disregarded,^(3,27)^ thus leading to a situation of a "life without value", which is also known as the "bare life".^(35)^ Byung-Chul Han, who built on the work of Agamben, described a person whose life may be subjected to death that is devoid of value and protection as well as stripped of any rights or significance as "homo sacer"; such a life is represented as "absolutely killable".^(35,36)^ This process of dehumanization further highlights the inherent vulnerability of critically ill patients.

Nevertheless, while such vulnerabilities must be recognized and addressed, this concept must be used with caution, as the concept of vulnerability may lead to paternalism and oppression, social control or the exclusion and stigmatization of vulnerable populations.^(28)^

### Prospective autonomy, relational autonomy, and vulnerability in the context of critical care

The vulnerabilities and difficulties associated with the exercise of autonomy by critically ill patients have led to increasing interest in the mechanisms of prospective autonomy, by which a person may make prospective decisions regarding future situations in which they may be incompetent to choose.^(37)^ Through the use of advance directive documents, such as living wills or healthcare durable power of attorney, a person may make decisions and formulate instructions regarding future medical treatments according to their own will, values and preferences, thus exerting their right to self-determination.^(25,37,38)^ The literature has reported that the use of living wills or durable power of attorney documents is associated with surrogate decision-making that can lead to care that is in line with patients' previously stated preferences.^(39)^ Nevertheless, the presence of advance directive documents in Brazil is rare, which may impede the use of those tools in clinical practice.^(40)^ Moreover, scholars have raised the concern that advance directives may pose ethical challenges in the acute setting, especially among patients who have not previously experienced disabling health conditions, who may thus not have had all the information necessary to support informed decision-making.^(37,41)^

High-stake decision-making in acute settings may exacerbate biases toward lower quality of life that are associated with potential disabilities, in which context healthy people usually perceive future limitations as worse than do people who actually live with such limitations.^(42)^ This "disability paradox" leads to a failure to consider one's ability to adapt to new disabilities, a tendency to undervalue one's capacity to cope with losses and a tendency to focus on the negative aspects of such a situation without accounting for the possibility of continued access to the "goods of life".^(42)^ Empirical data have supported the frequent tendency of individuals to fail to predict their quality of life after major life events accurately^(43)^ and confirmed the subjective well-being experienced by patients with disabilities after major illnesses.^(44-47)^ As such, in the absence of effective communication between health care professionals and patients or surrogates, decisions may be made on the basis of misconceptions, which may not be in the best interest of patients. Therefore, acute settings make it more challenging to guarantee patients' right to self-determination while reducing the risk of decision-making biases and mitigating the risk of paternalistic approaches on the part of health care providers.

**Figure 1:**
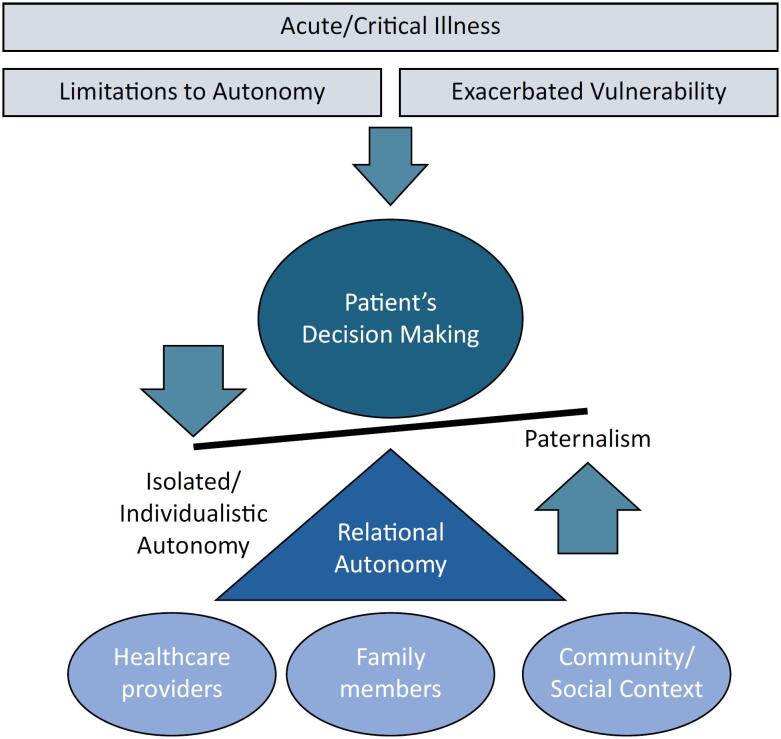
Schematic diagram of the relationships among autonomy, vulnerability and critical illness in the decision-making process among patients.

Thus, decisions may necessarily be made in a collective manner that involves the patient, surrogates, and health care professionals in a joint commitment and seeks to establish symmetric relations among all stakeholders^(26)^ (Figure 1). This approach has been supported by the frameworks of shared decision-making^(48,49)^ and substituted relational autonomy.^(50,51)^ which also focus on mechanisms by which the issues of prospective autonomy, vulnerabilities and autonomous decision-making can be addressed.^(19,27,52)^ These methods may help balance the principles of beneficence and respect for autonomy^(24)^ in light of their interdependence with vulnerability,^(27)^ thus making it impossible for healthcare providers to avoid their clinical responsibilities and preventing the inadvertent solitary delegation of decision-making to patients and surrogates in the absence of proper professional guidance, in which context health care professionals may abdicate their duty to make or recommend technical decisions by assigning this responsibility to patients or their surrogates.^(24,48,53)^

The ability of the relational autonomy model to establish a balance between isolated autonomy and paternalism is also illustrated.

Those frameworks rely on the premise that autonomy is not solitary; that is, autonomy is not exercised in a "social and cultural void"^(54)^ and is not binary ("all-or-nothing") but rather gradual.^(25)^ Furthermore, autonomy is more than the cognitive ability to make isolated decisions^(54)^ in the process of developing a relational, solidary form of autonomous decision-making.^(19)^ However, these methods entail certain risks, as they may still result in paternalistic attitudes on the part of health care professionals as a result of power and knowledge asymmetry, the stigmatization of vulnerabilities and potential conflicts with patients and their surrogates.^(28,51)^ Therefore, health care professionals play a more active role in the processes of understanding patients' preferences and guiding the decision-making process. As part of these processes, health care providers should aim to provide safe, evidence-based and patient-centered care, in which context decisions are made in a collaborative manner that takes into account not only the technical and biomedical aspects of care but also patients' biographies.^(48,55)^ Moreover, even in situations in which patients' preferences may not be well documented or well known, this approach may improve partnerships between health care providers and patients' surrogates in the decision-making process, which is likely to lead to better outcomes for both patients and caregivers.^(56)^ Furthermore, those collaborative approaches are supported by ethical and legal frameworks in Brazil,^(5,17,38,40,53)^ which promote respect for individual patient autonomy as well as the protection of vulnerabilities and the right to a dignified life while simultaneously trying to ensure that the most appropriate medical care is provided in line with the principles of beneficence and nonmaleficence as well as the goal of ensuring professional safety.

## CONCLUSION

Critically ill patients are vulnerable, and their autonomy may be disregarded, both because they may be subjected to inappropriate paternalistic actions and because they may be viewed as fully autonomous agents when they are not. To help overcome those challenges, the capacity to exercise autonomy must always be assessed accurately, and vulnerabilities must be acknowledged. Moreover, autonomous decision-making on the part of critically ill patients must not be viewed as a solitary, individualistic endeavor but rather as a relational ability, thus allowing patients to address their vulnerabilities and promote autonomy and engagement. This process should be constructed through a joint process among health care professionals, surrogates, and, when possible, patients themselves.
